# Progress of Veterinary Science

**Published:** 1887-07

**Authors:** 


### PROGRESS OF VETERINARY SCIENCE.

Whooping-Cough in a Cat.—In the British *Med. Jour.*, May 7, 1887, Mr. O. Bowen, of Liverpool, says: "For the past few weeks I have had under my care a little boy suffering from a more than usually severe attack of whooping-cough, and his mother informs me that for quite a fortnight their cat has had five or six distinct fits of coughing daily, similar in every respect to the boy's, which end after the expectoration of frothy mucus. The cat between the attacks is tolerably bright and active, though not so lively as she formerly was, and has considerably fallen away in condition. This is the first instance of a domestic animal becoming affected through, apparently, infection from a human being, which I can remember having observed, and it occurred to me that the fact may be of interest to others."

Bacteriology.—Among matters of special interest to those interested in Comparative Pathology were the lectures given this Spring by Professor Ramsey Wright, of University College. The course consisted of five lectures, which were delivered specially to veterinary students in the Ontario Veterinary College, but which were attended by medical men and veterinarians from different parts of the provinces. Professor Wright, who is a master of the subject—recognized as one of the best authorities on the continent in this department, gave a clear account of the various micro-organisms, with methods of cultivation. Practical demonstrations of "cultures" were given. The more important diseases produced by these organisms were touched upon more or less tully. The importance of the subject is so great, especially from the standpoint of comparative medicine, that no effort to advance it can be considered as superfluous.

The Cow as a Source of Scarlet Fever.—Unquestionably there is much to be gained by studying any possible connection there may be between man's diseases and those of his brute companions. Such a course has been pursued in and about New York at times during the past few years, especially with reference to scarlet fever, but the public statements made by those who have prosecuted the inquiries do not seem to have excited quite so much attention as might have been expected. Toward the close of the year 1885, Dr. E. Klein, of London, undertook a bacteriological investigation in this direction, and he gives an account of his observations in a paper contributed to the "Proceedings of the Royal Society," vol. xlii, No. 253, containing among others, the proceedings of the meeting of March 3, 1887. Dr. Klein satisfied himself of the morphological identity of a micrococcus found in the blood of human scarlet-fever patients with that obtained and cultivated from certain cows affected with a similar disease. He also inoculated eight calves with cultivations of the micrococcus of scarlet fever in the human subject, and in all of them a disease was developed that seemed identical, as to both the cutaneous and the visceral lesions, with an affection produced in other calves by inoculation from the cows in question. Dr. Klein thinks it is evident from these observations that the danger of scarlatinal infection from the disease in the cow is real, and that toward the study and careful supervision of this bovine disease all efforts ought to be directed, in order to check the spread of scarlet fever in man, as well as in the interests of agriculturists.—*N. Y. Medical Journal.*

EARTH-WORMS AS CARRIERS OF ANTHRAX-POISON. (VON BOLLINGER).—Robert Koch, in a series of experiments, proved Pasteur to be mistaken in his theory that earth-worms, together with the soil adhering to them, took up the spores of anthrax and brought them to the surface of the earth. One of his experiments consisted in infecting a number of mice with these worms finely rubbed up, whereupon it was found that only one of the animals died of anthrax. Fely, who had performed a number of similar experiments, found that guinea pigs died of anthrax after having been infected with particles of dried earth-worms. Bollinger experimented upon twenty squirrels, white rats and guinea pigs, using 72 worms from the most celebrated anthrax districts of the Bavarian Alps, which he rubbed up, each separately, in sterilized water. Among 21 worms one only was found to produce anthrax, from which he naturally concluded that 5 per cent. of earth-worms contain anthrax poison. Johne, however, draws a different conclusion from Bollinger's experiments, which would go to show that out of 72 only one worm contained anthrax poison. This would also substantiate Koch's view that earth-worms are very poor carriers of anthrax.—*Weekly Medical Review.*

ACTINOMYCOSIS IN MAN.—Up to now 75 authentic cases of actinomycosis hominis have been observed. In regard to the etiology, Moosbrugger supposes that with man, as well as animals, the cause can be traced back to the consumption of grain, such as barley, or the inhalation of the specific germs into the respiratory passages. A direct inoculation of the disease from the animal upon man, or infection by eating sick meat, although not impossible, appears rather improbable. Suppuration, although frequently present, is not always necessary, and generally only found in older cases. The prognosis depends upon the seat of the disease, deep affections, where operative interference is impossible, being the more serious.—*Weekly Medical Review.*

